# Stepwise acupuncture exploration followed by ultrasound-guided needle-knife release combined with local infiltration for chronic intercostal muscle–related myofascial pain: A case report

**DOI:** 10.1097/MD.0000000000051333

**Published:** 2026-10-23

**Authors:** Yuyang Ou, Dan Li, Guanghan Li, Hui Li

**Affiliations:** a Department of Acupuncture, Third Affiliated Hospital of Beijing University of Chinese Medicine, Beijing, China; b Department of Ultrasound Medicine, China-Japan Friendship Hospital, Beijing, China; c Department of Acupuncture, China-Japan Friendship Hospital, Beijing, China.

**Keywords:** acupotomy, case report, intercostal muscle, myofascial pain, ultrasound guidance

## Abstract

**Rationale:**

Intercostal muscle–related myofascial pain can be difficult to localize when superficial tenderness is absent because the muscles lie deep to the thoracic musculature and close to the pleura. We report a case in which stepwise muscle-specific acupuncture provided ancillary localization information before targeted treatment.

**Patient concerns:**

A 73-year-old man presented with a 3-year history of persistent right posterolateral chest wall heaviness and dull pain, predominantly involving the fourth to tenth intercostal spaces. The resting numerical rating scale (NRS) score was 8, and no definite superficial tender point was identified.

**Diagnoses:**

Clinical and imaging evaluations excluded more plausible cardiopulmonary, pleural, osseous, spinal, herpetic, or neuropathic causes. More than 1 month of sequential acupuncture targeting the trapezius, rhomboid, multifidus, erector spinae, and serratus muscles produced no meaningful improvement. When exploration reached the suspected deep intercostal layer, ultrasound-guided acupuncture reproduced the habitual pain and produced mild relief. This response was interpreted as hypothesis-generating localization information rather than confirmatory evidence and contributed to a provisional diagnosis of intercostal muscle–related myofascial pain.

**Interventions:**

The patient underwent 4 weekly sessions of ultrasound-guided needle-knife release combined with local infiltration of 0.25% lidocaine and compound betamethasone. The intercostal anatomy and needle tip were monitored in real time using an out-of-plane approach.

**Outcomes:**

The NRS score decreased from 8 to 5 immediately after the first treatment and further decreased to 1 one week after the fourth treatment. Pain completely resolved at the 1-, 3-, and 6-month follow-ups, with no treatment-related adverse events.

**Lessons:**

Stepwise muscle-specific acupuncture may provide ancillary localization information when conventional examination cannot identify a deep chest wall pain generator. Ultrasound-guided needle-knife release combined with local infiltration targeting the suspected intercostal muscle was associated with sustained improvement and may represent a potential treatment approach for selected patients with deep intercostal muscle–related myofascial pain.

## 1. Introduction

Musculoskeletal chest wall pain comprises a heterogeneous group of disorders arising from the ribs, joints, muscles, and fascial tissues. Pain originating from the intercostal muscles and associated fascia may present as persistent soreness, heaviness, tightness, or dull pain and may overlap clinically with cardiopulmonary, pleural, osseous, spinal, and neuropathic disorders.^[1–4]^ Intercostal muscle–related myofascial pain is especially difficult to recognize when no superficial tender point is present.

The intercostal muscle complex occupies the space between adjacent ribs and lies immediately superficial to the pleura, while the intercostal neurovascular bundle courses within the deeper intercostal plane.^[3]^ Surface palpation may therefore be unable to distinguish an intercostal pain generator from overlying rhomboid, latissimus dorsi, or paraspinal tissues. No laboratory or imaging test confirms an intercostal myofascial source. Clinical attribution is inferential and should integrate pain quality and distribution, mechanical provocation, reproduction of the patient’s familiar pain, and assessment for more plausible alternative causes.^[1,2]^

Conservative management of myofascial pain may include activity modification, analgesics, physical therapy, manual therapy, acupuncture, dry needling, and local injection.^[5,6]^ A needle-knife, also termed acupotomy, is a percutaneous instrument with a small blade-like tip used to mechanically release selected soft tissues. Trials and reviews in other myofascial and musculoskeletal conditions suggest potential analgesic and functional benefits, including with ultrasound guidance, but the methods and evidence quality are heterogeneous and evidence specific to intercostal muscle–related pain is lacking.^[7–11]^ Because the target intercostal plane is thin and adjacent to the pleura and neurovascular structures, ultrasound visualization may assist anatomical targeting and procedural risk management.^[10–12]^

We report a 73-year-old man with chronic right posterolateral chest wall pain and no superficial tender point. Before ultrasound-guided needle-knife treatment, the patient underwent more than 1 month of sequential acupuncture targeting individual muscle layers. The trapezius, rhomboid, multifidus, erector spinae, and serratus muscles were treated in turn without meaningful improvement. When the exploration reached the deepest suspected intercostal muscle layer, ultrasonography was introduced to identify the deep intercostal anatomy and guide acupuncture at this level. Needling reproduced a deep aching sensation similar to the patient’s habitual pain and was followed by mild symptomatic relief. This layer-dependent response was used as hypothesis-generating ancillary localization information, and the same anatomically localized intercostal muscle was subsequently targeted with ultrasound-guided needle-knife release combined with local infiltration. This report describes the diagnostic uncertainty, sequential therapeutic exploration, ultrasound-guided procedural safeguards, and six-month clinical course. This case report was prepared in accordance with the CARE guidelines.

## 2. Case report

A 73-year-old man presented to the outpatient acupuncture clinic on March 19, 2025, with a 3-year history of right-sided chest wall pain. Approximately 6 months before the onset of pain, in September 2021, he had been diagnosed with Coronavirus Disease 2019 (COVID-19). During the acute phase of infection, his main symptoms included fever, cough, chest tightness, dyspnea, and fatigue, which gradually resolved after symptomatic treatment. Six months later, localized right-sided chest wall pain gradually developed and subsequently persisted. The painful area extended approximately from the level of the the third to the tenth ribs, predominantly involving the 4th to 10th intercostal spaces, from the paraspinal region medially to near the posterior axillary line laterally (Fig. 1B). The pain was described as a diffuse, persistent dull pain over the right posterolateral chest wall, accompanied by a pronounced sensation of heaviness, as though an additional layer were weighing on the chest wall. The pain was not burning or electric shock–like and did not radiate in a dermatomal pattern. The resting numerical rating scale (NRS) score was 8. The pain was continuous, was not relieved by rest, and was not aggravated by deep breathing or trunk rotation. It interfered with the patient’s sleep and daily activities. His medical history included cerebral infarction and hyperlipidemia. He was receiving long-term aspirin therapy and had a known allergy to tetracycline. There was no history of herpes zoster involving the painful area. On November 26, 2024, he attended another hospital and received oral traditional Chinese medicine and topical flurbiprofen cataplasms, with no appreciable improvement.

**Figure 1.**
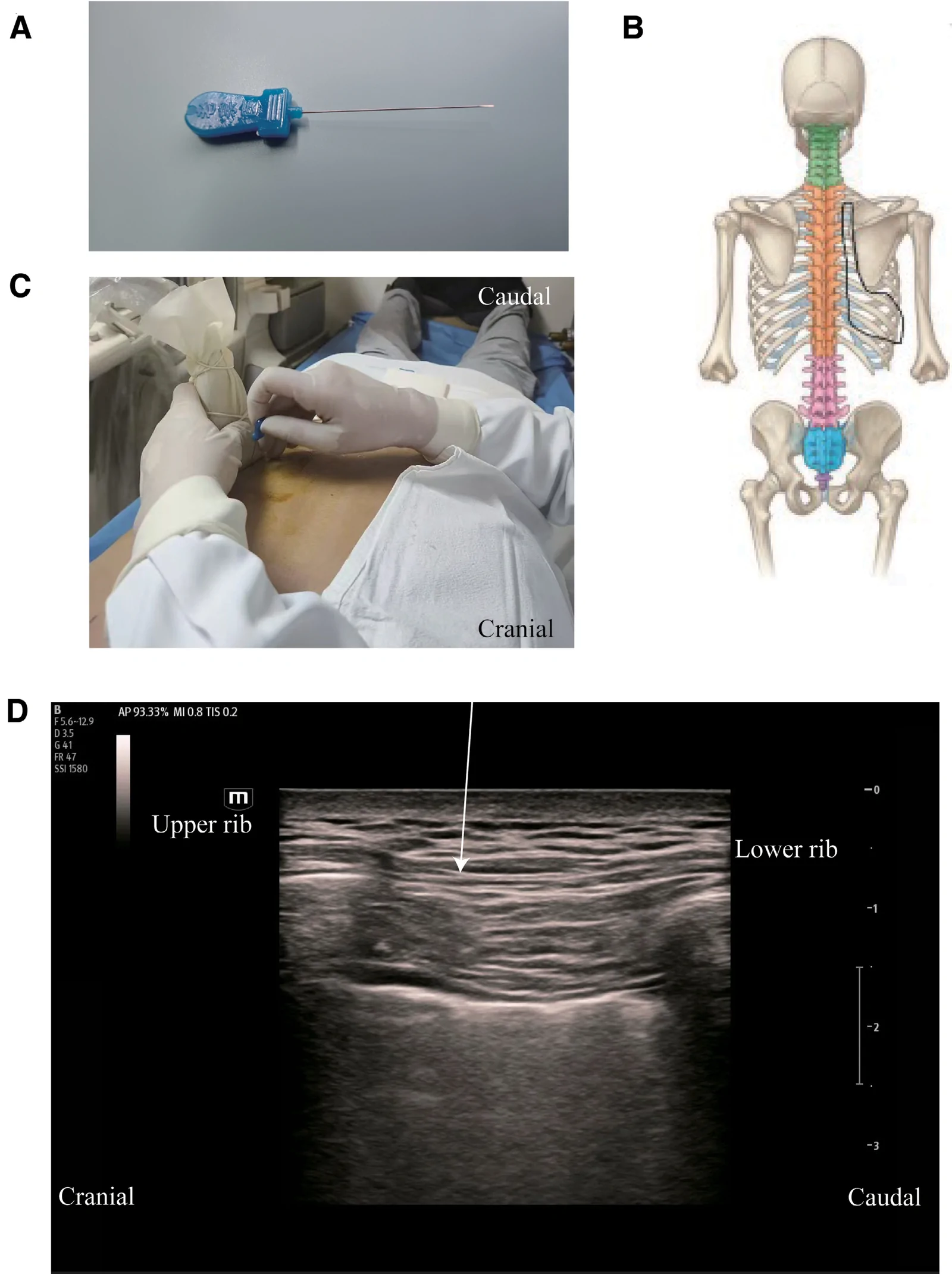
Clinical presentation and ultrasound-guided needle-knife release for chronic intercostal muscle–related myofascial pain. (A) The 0.4 mm × 40 mm needle-knife used during the procedure. (B) Schematic illustration of the distribution of pain over the patient’s right posterolateral chest wall. The affected area, outlined in black, extended from the level of the third rib to the tenth rib. The anatomical schematic was created by the authors. (C) Clinical photograph showing the patient’s position during the ultrasound-guided procedure. A high-frequency linear-array transducer was positioned perpendicular to the long axis of the ribs. (D) Oblique sagittal ultrasound image of the chest wall showing the adjacent ribs in short-axis view. The white arrow indicates the dynamically confirmed position of the needle-knife tip within the intercostal muscle complex. Because an out-of-plane approach was used, tip location was established by incremental needle movement and real-time transducer adjustment rather than by the static image alone. The continuous hyperechoic line deep to the intercostal muscle complex represents the pleura. The left side of the image corresponds to the cranial direction, and the right side to the caudal direction.

Available evaluation included electrocardiography, chest radiography, pulse oximetry, thoracic magnetic resonance imaging (MRI), and targeted history and physical examination. These assessments did not reveal any acute cardiopulmonary abnormality, pleural effusion, pneumothorax, evident rib fracture, active pulmonary infection, herpes zoster–related disorder, or other clear cause of the chronic chest wall pain. Thoracic MRI showed ligamentum flavum thickening and a mild T9–T10 intervertebral disc protrusion. Although the T9–T10 intervertebral disc protrusion partially overlapped with the lower portion of the painful area, several clinical features argued against it as the primary pain generator. The patient’s symptoms involved a broad distribution from the 3rd to 10th ribs rather than a localized lower thoracic radicular pattern, and he did not exhibit neuropathic pain characteristics, dermatomal radiation, or sensory abnormalities. Therefore, the mild T9–T10 disc protrusion was considered insufficient to explain the overall clinical presentation.

Physical examination revealed no definite localized tender point over the right posterolateral chest wall. No sensory abnormality was identified over the affected area. Palpation of the 4th to 10th intercostal spaces, adjacent rib surfaces, paraspinal region, and overlying soft tissues failed to consistently reproduce or localize the patient’s familiar persistent heavy pain. No marked tenderness was identified over the thoracic spinous processes, facet joints, costovertebral joints, or costotransverse joints. Because the painful area was extensive and conventional surface palpation yielded no clear positive localizing sign, physical examination alone could not identify the pain-generating tissue or its anatomical layer.

Because physical examination did not provide a clear tissue-level target, a stepwise therapeutic exploration with acupuncture was undertaken before considering ultrasound-guided needle-knife treatment. From March to early May 2025, over a period of more than 1 month, stepwise therapeutic exploration with acupuncture was performed, targeting the trapezius, rhomboid, multifidus, erector spinae, and serratus muscles sequentially, one muscle target at a time. These treatments produced no meaningful improvement in the patient’s habitual posterolateral chest wall heaviness and dull pain. When the stepwise exploration subsequently reached the deepest suspected intercostal muscle layer, ultrasonography was introduced to identify the intercostal muscle complex, rib cortices, pleural line, and visible vascular structures and to guide acupuncture at this level. Ultrasound-guided needling of the suspected intercostal muscle reproduced the patient’s familiar deep aching pain, representing a concordant pain response, and the patient reported mild but noticeable relief of his original symptoms after treatment. The combination of concordant pain reproduction and the partial therapeutic response, in contrast to the absence of meaningful response to treatment of the more superficial muscle layers, increased our clinical suspicion that the intercostal muscle and its associated fascial plane might represent the principal pain-generating region; however, these observations were interpreted only as hypothesis-generating localization information rather than confirmatory evidence, because sequential acupuncture is not a validated diagnostic test. Accordingly, a provisional diagnosis of intercostal muscle–related myofascial pain was made. This clinically suspected deep layer, anatomically localized under ultrasound, was subsequently selected as the target for needle-knife release combined with local infiltration.

The patient was placed in the prone position. Ten treatment targets were selected within the right 4th to 10th intercostal spaces according to the distribution of pain and the deep intercostal muscle localized during the preceding stepwise acupuncture exploration under ultrasound guidance. After routine skin preparation and sterile draping, the target intercostal spaces were examined using a high-frequency linear-array transducer (Mindray TEX20T, 5.6–12.9 MHz). The transducer was positioned perpendicular to the long axis of the ribs and adjusted to visualize the superficial chest wall tissues, intercostal muscle complex, rib cortices with posterior acoustic shadowing, pleural line, and any visible vascular structures (Fig. 1C).

Local infiltration was performed first. A 0.9 mm × 38 mm injection needle (BD, USA) was introduced using an ultrasound-guided out-of-plane approach and advanced into the target intercostal muscle layer. The total injectate volume was 10 mL and consisted of 1.25 mL of 2% lidocaine, 0.5 mL of compound betamethasone injection containing 2.5 mg of betamethasone dipropionate and 1 mg of betamethasone sodium phosphate, and normal saline added to a final volume of 10 mL. The final lidocaine concentration was 0.25%. Approximately 1 mL of the solution was deposited at each of the 10 treatment targets under ultrasound observation.

Needle-knife release was subsequently performed in the same target regions using a 0.4 mm × 40 mm needle-knife (Laozongyi Medical Devices Co., Ltd., Nanchang, China) (Fig. 1A). The needle-knife was introduced from the center of the transducer using an out-of-plane approach and advanced in small increments toward the intercostal muscle complex. Because a punctate hyperechoic reflection in an out-of-plane view may represent either the needle tip or a transverse section of the shaft, the presumed tip position was repeatedly confirmed by dynamic transducer adjustment, subtle needle movement, and concordant movement of the hyperechoic point (Fig. 1D; Supplemental Video 1, Supplemental Digital Content 1). The release procedure was performed within the target intercostal muscle and fascial plane. Visible vascular structures were avoided, and advancement was stopped when a visible distance between the needle-knife and the pleural line remained.

After completion of the procedure, the pleural line and lung sliding were reassessed using ultrasound, and no immediate sonographic sign of pneumothorax was identified. After withdrawal of the needle-knife, local pressure was applied for hemostasis, and the puncture sites were covered with sterile dressings. Because the patient was receiving long-term aspirin therapy, bleeding risk was assessed before treatment, visible vascular structures were carefully avoided, and the puncture sites were monitored for bleeding and hematoma formation. The patient tolerated the procedure well. No dyspnea, dizziness, bleeding, subcutaneous hematoma, vasovagal reaction, or other immediate adverse event was observed.

The patient subsequently received 4 weekly treatment sessions on May 5, 12, 19, and 26, 2025. The treatment area and procedure during the second session were identical to those used during the first session. During the 3rd and 4th sessions, the treatment targets were adjusted according to the distribution of residual pain and were primarily confined to 4 targets within the right 8th to 10th intercostal spaces. Both local infiltration and needle-knife release were continued. Approximately 1 mL of injectate was administered at each target, resulting in a total injectate volume of approximately 4 mL per session. The final lidocaine concentration remained 0.25%, and 0.5 mL of compound betamethasone injection was included in the injectate during each session.

At the follow-up assessment 1 week after completion of the 4 treatment sessions, the patient reported further improvement in right-sided chest wall pain and a marked reduction in pain during daily activities compared with baseline. The resting NRS score was 1. At the 1-, 3-, and 6-month follow-up assessments, the chest wall pain had completely resolved, with an NRS score of 0 at each time point and restoration of normal sleep and daily activities. No additional analgesic or other medication specifically for chest wall pain was used during follow-up. No delayed adverse events were observed. The patient’s clinical course and interventional outcomes are summarized in Table 1.

**Table 1:** Timeline of the patient’s clinical course and interventional outcomes.

Date/ time	Clinical event/ Intervention	Clinical findings & symptoms	Efficacy/ Outcomes (NRS)
September 2021	COVID-19 infection	Acute symptoms (fever, cough, chest tightness, etc) resolved after symptomatic treatment	No chest wall pain was reported during the acute infection.
March 2022	Chest wall pain onset	Localized right posterolateral chest wall pain gradually developed and persisted thereafter	N/A
November 26, 2024	Initial external treatment	Visited another hospital.	Received oral traditional Chinese medicine and topical flurbiprofen cataplasms; no significant relief.
March 19, 2025	Initial presentation and evaluation	Presented at our clinic with a 3-yr history of right chest pain.	Pretreatment NRS: 8.
March 2025	Diagnostic workup	Underwent ECG, chest X-ray, and thoracic MRI.	No more plausible cardiopulmonary, pleural, osseous, herpetic, or thoracic radicular cause was identified.
March–early May 2025	Stepwise acupuncture exploration	Trapezius, rhomboid, multifidus, erector spinae, and serratus muscles were treated sequentially, one target at a time, without meaningful improvement. When exploration reached the deepest suspected intercostal muscle layer, ultrasound was introduced to identify the deep intercostal anatomy and guide acupuncture. Needling reproduced a deep aching sensation similar to the patient’s habitual pain and was followed by mild symptomatic relief.	The layer-dependent response provided hypothesis-generating localization information and prompted provisional selection of the deep intercostal muscle as the treatment target.
May 5, 2025	1st ultrasound-guided needle-knife treatment	The previously localized deep intercostal muscle was targeted under ultrasound guidance; local infiltration and needle-knife release were performed at 10 targets in the right 4th–10th intercostal spaces.	Immediate relief; NRS 8→5. No adverse events.
May 12, 2025	2nd ultrasound-guided treatment	Treatment was identical to the first session.	Further improvement; well tolerated.
May 19 and 26, 2025	3rd and 4th ultrasound-guided treatments	Treatment targets were adjusted according to the residual painful area and were primarily confined to 4 targets within the right 8th–10th intercostal spaces.	Progressive improvement; both sessions were well tolerated, with no pneumothorax, vascular injury, or other adverse events.
1 wk; 1, 3, and 6 mo after treatment	Follow-up	Pain markedly reduced at 1 wk and subsequently resolved completely.	NRS 1 at 1 wk; NRS 0 at 1, 3, and 6 mo. No delayed adverse events.

COVID-19 = Coronavirus Disease 2019, ECG = electrocardiography, MRI = magnetic resonance imaging, NRS = numerical rating scale.

### 2.1. Patient perspective

The patient reported that repeated acupuncture directed at the more superficial and intermediate muscle targets had not meaningfully changed his habitual chest wall symptoms. When ultrasound-guided acupuncture was subsequently directed to the deepest suspected intercostal region, he experienced a deep aching sensation similar to his usual pain and noticed mild relief of his original symptoms after treatment. He subsequently perceived progressive improvement after the ultrasound-guided treatment sessions and reported complete resolution of the pain symptoms, accompanied by restoration of normal daily activities and sleep, according to patient report at the six-month follow-up. He did not report any treatment-related concerns or delayed adverse effects.

## 3. Discussion

This case illustrates 2 clinically relevant aspects of the management of chronic chest wall pain: localization of a possible deep intercostal soft-tissue pain generator when conventional examination provides no clear superficial target, and the use of ultrasound guidance to improve the anatomical precision and procedural safety of treatment within a thin intercostal plane adjacent to the pleura and neurovascular structures. In the present patient, these 2 aspects were closely linked. Sequential muscle-specific acupuncture was used to explore the possible pain-generating layer, and when the exploration reached the suspected deep intercostal muscle layer, ultrasound guidance was used to identify this anatomical plane and guide needling. The findings obtained at this level subsequently provided the basis for ultrasound-guided needle-knife release combined with local infiltration targeting the same intercostal muscle.

The first challenge was determining the likely source of the pain. The patient had chronic, unilateral, persistent heaviness and dull pain predominantly involving the right 4th to 10th intercostal spaces. The pain was not burning, electric shock–like, or dermatomally radiating, and no sensory abnormality was identified. Available clinical and imaging evaluations did not reveal a cardiopulmonary, pleural, osseous, spinal, or neuropathic abnormality that adequately explained the location and characteristics of the symptoms.^[2,4,13]^ Thoracic MRI showed ligamentum flavum thickening and a mild T9–T10 intervertebral disc protrusion. Although the T9–T10 level partially overlapped with the lower portion of the painful area, the extensive symptom distribution and absence of typical thoracic radicular features made this finding less likely to represent the primary pain generator. Given the chronic and persistent nature of the symptoms and the absence of a more plausible alternative explanation, a soft-tissue source of the chest wall pain was considered. The temporal relationship with the previous COVID-19 infection also deserves consideration. Persistent musculoskeletal symptoms, including myalgia and chest wall pain, have been reported after SARS-CoV-2 infection.^[14]^ In this patient, chest wall pain developed approximately 6 months after COVID-19 infection, suggesting a possible temporal association. However, no chest wall pain was present during the acute infection, and clinical or imaging evaluations did not identify specific findings supporting a direct causal relationship. Therefore, previous COVID-19 infection was considered a potential contributing context during clinical assessment.

Identifying the specific soft-tissue layer was more difficult. Classic descriptions of myofascial pain frequently emphasize a palpable tender point or taut band and reproduction of the patient’s familiar pain.^[1,15]^ In this patient, however, no definite superficial tender point was identified. The intercostal muscles are relatively thin and lie deep to the overlying posterolateral thoracic musculature, making them difficult to assess by surface palpation alone. We therefore used sequential acupuncture to explore the possible muscular source layer by layer. Treatment of the trapezius, rhomboid, multifidus, erector spinae, and serratus muscles did not meaningfully change the patient’s habitual symptoms. When exploration subsequently reached the suspected intercostal muscle layer, ultrasound was used to identify the deep intercostal anatomy and guide acupuncture at this level. Needling of this layer reproduced the patient’s familiar deep aching pain, resulting in a concordant pain response, and was followed by mild but noticeable symptomatic relief. In contrast to the absence of meaningful responses at the more superficial muscular levels, these findings shifted our clinical focus toward the intercostal muscle and associated fascial plane. Importantly, pain reproduction together with subsequent relief was regarded only as hypothesis-generating information and did not establish the diagnosis.

Once the deep intercostal soft-tissue plane had been provisionally selected, the next clinical question was whether conventional acupuncture alone was sufficient or whether a more mechanically directed intervention was warranted. Acupuncture and needle-knife treatment both involve percutaneous needling, but their intended mechanical actions differ. Conventional acupuncture primarily provides needle-based stimulation without deliberate tissue cutting, whereas the bladelike tip of the needle-knife permits controlled mechanical release within selected soft-tissue planes. In chronic myofascial pain, fascial restriction, impaired tissue gliding, altered local mechanical loading, and, in some conditions, fibrotic or adhesion-like changes have been proposed as contributors to persistent nociceptive input. Mechanical release has therefore been hypothesized to restore tissue mobility and reduce abnormal local mechanical stress, with secondary effects on local circulation, inflammatory signaling, and nociception.^[16]^

In this patient, more than 1 month of acupuncture directed sequentially at the superficial and intermediate muscular layers produced no meaningful improvement, while ultrasound-guided acupuncture of the suspected intercostal layer reproduced the habitual pain and resulted in only mild symptomatic relief. Given the three-year duration and high baseline pain intensity, this limited response was considered insufficient for symptom control. Needle-knife treatment was therefore selected not because the acupuncture response established the diagnosis, but because it offered controlled mechanical release of the suspected intercostal soft-tissue plane, an action that conventional acupuncture was not designed to provide.

The mechanical releasing capability of the needle-knife also makes precise anatomical control particularly important when it is used in the intercostal region. Unlike more superficial myofascial targets, the intercostal muscle complex occupies a relatively narrow anatomical space between adjacent ribs and lies immediately superficial to the pleura. The intercostal neurovascular structures also course within the deeper intercostal plane. Accurate identification of the intended muscle layer and precise control of needle depth are, therefore, particularly important when interventions are performed in this region. In the present case, ultrasound was first incorporated during acupuncture of the suspected intercostal layer and was subsequently used throughout the definitive treatment. It allowed direct visualization of the intercostal muscle complex, rib cortices, pleural line, and visible vascular structures, enabling the clinically suspected deep layer to be treated as a clearly defined anatomical target rather than by surface estimation alone.

Ultrasound guidance was used throughout both local infiltration and needle-knife release to improve targeting precision and procedural risk control. With the out-of-plane approach used in this case, a punctate hyperechoic reflection may represent either the needle tip or a transverse section of the shaft; therefore, a static ultrasound image alone cannot reliably confirm the actual tip position.^[12]^ The presumed tip position was repeatedly verified through small incremental advancement, dynamic transducer adjustment, and subtle needle movement with concordant displacement of the hyperechoic point. Visible vascular structures were avoided, and advancement was stopped while a visible distance from the pleural line remained. The pleural line and lung sliding were reassessed immediately after treatment. These procedures enabled the infiltration needle and needle-knife to be directed toward the intended intercostal muscle and fascial plane while maintaining continuous visualization of adjacent pleural and vascular structures. Ultrasound guidance was not considered an independent therapeutic modality in this case; rather, it facilitated anatomical identification of the suspected intercostal soft-tissue plane, improved the precision of treatment delivery, and may, therefore, have contributed indirectly to the clinical response through more accurate targeting, while also reducing the risks of pleural and vascular injury.

Needle-knife treatment has been investigated in several other myofascial and musculoskeletal pain conditions, including randomized trials and systematic reviews.^[7–10]^ Existing evidence suggests potential analgesic and functional benefits, although heterogeneity in target tissues, treatment techniques, comparators, and methodological quality limits direct extrapolation to intercostal muscle–related pain.^[8,9]^ In the present case, needle-knife release was combined with local infiltration of lidocaine and compound betamethasone rather than used as an isolated intervention. The patient experienced an immediate decrease in NRS score from 8 to 5 after the first session, followed by progressive improvement during 4 weekly treatment sessions, with an NRS score of 1 one week after treatment and 0 at the 1-, 3-, and 6-month follow-ups. This sustained clinical response suggests that accurately targeted treatment of the suspected intercostal soft-tissue plane may have clinical value. The observed improvement may have resulted from the combined effects of needle-knife release and local corticosteroid administration, which represent the major therapeutic components of the intervention. Although local anesthetic injection with lidocaine may also provide analgesic effects in some pain conditions, its individual contribution in this case is likely limited. Because these components were delivered as a combined intervention, the specific contribution of each component cannot be established in this case.Given the 3-year persistence of symptoms before treatment and the sustained pain resolution through 6 months of follow-up, natural symptom fluctuation and regression to the mean appear less likely to fully explain the observed clinical course, although they cannot be completely excluded in a single case.

Procedural risk was particularly relevant because of the proximity of the treatment target to the pleura and intercostal vessels and because the patient was receiving long-term aspirin therapy. Bleeding risk was assessed before treatment, visible vascular structures were avoided under ultrasound guidance, needle manipulation was carefully controlled, and local compression and postprocedural observation were performed. No bleeding, subcutaneous hematoma, pneumothorax, dyspnea, vasovagal reaction, or delayed treatment-related adverse event was observed during the treatment course or follow-up. Nevertheless, adverse events after needle-knife treatment have been reported in prospective studies.^[11]^ The uneventful procedural course in this patient demonstrates the clinical feasibility of ultrasound-guided treatment of the deep intercostal muscle and highlights the value of direct visualization for precise targeting and risk management. Appropriate knowledge of intercostal ultrasound anatomy and reliable needle-tip control remain essential when interventions are performed in this region.

Several limitations should be considered. First, this report describes a single patient and therefore cannot establish the diagnostic validity or therapeutic efficacy of this approach. Although the patient reported complete resolution of pain symptoms together with restoration of sleep and daily activities during follow-up, activity-related pain was not systematically quantified using a separate pain scale, and validated functional or quality-of-life instruments and objective assessments were not collected. Future prospective studies should incorporate standardized functional instruments and objective measures, such as pressure pain threshold and functional performance assessments, to better evaluate clinical outcomes. Second, localization of the intercostal layer was partly based on sequential acupuncture over more than 1 month. Although the differences in concordant pain reproduction across muscular layers contributed to target selection, cumulative or delayed effects of previous acupuncture, natural symptom fluctuation, expectation, and differences in needling depth or stimulation cannot be completely excluded. Future cases should separate hypothesis-generating localization from independent diagnostic validation. After provisional localization of the suspected intercostal level, a standardized ultrasound-guided low-volume local-anesthetic diagnostic intercostal block could be performed using predefined criteria for short-term relief of the habitual pain. Where feasible and ethically acceptable, a blinded placebo-controlled or repeated confirmatory design could further reduce expectation and nonspecific treatment effects. Such diagnostic testing should be performed separately from needle-knife release and corticosteroid injection so that the localization assessment is not confounded by the definitive intervention. Third, the definitive intervention combined needle-knife release, lidocaine, and corticosteroid, so the relative contribution of each treatment component cannot be determined. Future studies should further evaluate standardized methods for identifying intercostal muscle–related pain and systematically investigate the accuracy, safety, and clinical effectiveness of ultrasound-guided targeted treatment. Because acupuncture and needle-knife procedures are technique-dependent interventions, future multicenter and multi-operator studies with standardized procedural protocols and reporting of operator qualifications will be important to improve reproducibility and comparability.

In conclusion, this case illustrates that stepwise muscle-specific acupuncture may provide ancillary localization information when a deep chest wall pain generator cannot be identified by conventional examination. Ultrasound-guided needle-knife release combined with local infiltration targeting the deep intercostal muscle was associated with sustained clinical improvement, while ultrasound guidance may facilitate anatomical targeting and procedural risk control. These findings are hypothesis-generating and require confirmation in larger studies using standardized diagnostic and treatment protocols.

## Author contributions

**Conceptualization:** Dan Li.

**Data curation:** Yuyang Ou.

**Investigation:** Guanghan Li, Hui Li.

**Supervision:** Hui Li.

**Visualization:** Yuyang Ou.

**Writing – original draft:** Yuyang Ou.

**Writing – review & editing:** Dan Li, Hui Li.


